# Rare, late onset of immune checkpoint inhibitor-induced type 1 diabetes mellitus in a patient with small-cell lung cancer treated with serplulimab: a case report and review of the literature

**DOI:** 10.1186/s13256-023-04248-7

**Published:** 2024-01-22

**Authors:** Peng Ning, Shilan Liu, Hongyi Cao

**Affiliations:** 1 Department of Endocrine and Metabolism, Chengdu Fifth People’s Hospital, Cancer Prevention and Treatment Institute of Chengdu (The Second Clinical Medical College, Affiliated Fifth People’s Hospital of Chengdu University of Traditional Chinese Medicine), Chengdu, China; 2 Respiratory and Critical Care Medicine, Chengdu Fifth People’s Hospital, Cancer Prevention and Treatment Institute of Chengdu (The Second Clinical Medical College, Affiliated Fifth People’s Hospital of Chengdu University of Traditional Chinese Medicine), Chengdu, China

**Keywords:** Small-cell lung cancer, Serplulimab, Immune-checkpoint inhibitor–induced type 1 diabetes mellitus, Late-onset adverse events, Treatment management, Case report

## Abstract

**Background:**

As a newly approved immune checkpoint inhibitor in China, serplulimab has been widely used in the immunotherapy of tumors. However, the immune-related adverse events of immune checkpoint inhibitors should not be ignored. Although immune checkpoint inhibitor-induced type 1 diabetes mellitus is a rare complication, it may cause diabetic ketoacidosis and endanger the lives of patients.

**Case presentation:**

This case report describes a 55-year-old male of Han nationality from China diagnosed with small-cell lung cancer with multiple metastases who experienced an adverse event of type 1 diabetes mellitus 68 weeks after receiving serplulimab therapy. The patient presented with typical symptoms of diabetic ketoacidosis, including severe thirst, nausea, vomiting, deep respirations, and stupor. Despite the absence of diabetes-related autoantibodies, the patient had extremely low levels of insulin and C-peptide release. Other potential causes of diabetes were ruled out, confirming the condition as serplulimab-induced immune checkpoint inhibitor-induced type 1 diabetes mellitus. After aggressive treatment to correct diabetic ketoacidosis, the patient’s blood glucose levels stabilized and symptoms of diabetes improved significantly, although long-term insulin maintenance therapy was necessary.

**Conclusion:**

This case highlights a rare, late-onset adverse event of immune checkpoint inhibitor-induced type 1 diabetes mellitus that may be overlooked during treatment with serplulimab. The monitoring of blood glucose levels and early signs and symptoms of diabetes cannot be relaxed at the late stage of treatment, even if patients do not have elevated blood glucose levels before and during the middle stage of treatment.

## Introduction

With an increase in the clinical application of tumor immunotherapy, adverse reactions caused by immune checkpoint inhibitors (ICIs) are becoming increasingly common [1–3]. Adverse reactions caused by ICIs may involve multiple endocrine glands, and major diseases include thyroid dysfunction, hypophysitis, adrenal cortex dysfunction, and type 1 diabetes mellitus (T1DM) [4]. Among the adverse reactions, T1DM is extremely rare. The largest insurance claim database study of more than 30,000 people in the USA found that the incidence of ICI-induced T1DM (ICI-T1DM) was 0.86% [5]. In addition, according to literature statistics, the disease onset in 69% of patients is with diabetic ketoacidosis (DKA) [6]. Failure to promptly diagnose this condition can seriously endanger the lives of patients. Serplulimab (HLX10) is a novel ICI and the first innovative recombinant humanized monoclonal antibody against programmed cell death 1 (PD-1), independently developed by Henlius Biotech Limited. In March 2022, the US Food and Drug Administration (FDA) granted orphan drug designation to serplulimab for the treatment of patients with small-cell lung cancer (SCLC), thereby becoming the first approved anti-PD-1 monoclonal antibody for the first-line treatment of SCLC worldwide [7]. It was also approved by the National Medical Products Administration of China [8, 9] in the same month and can also be used to treat microsatellite instability-high solid tumors and squamous non-small-cell lung cancer.

Here, we present the case report of a patient with SCLC with multiple metastases who developed severe DKA and β cell failure 68 weeks after the initiation of serplulimab therapy, which was much later than the median time of 10 weeks for ICI-T1DM [5] and extremely rare in clinical practice. We hope that this case report and literature review will serve as a reference in the diagnosis and treatment of the rare, late-onset adverse events of ICI-T1DM and capture the attention of clinicians and healthcare professionals.

## Case presentation

**Fig. 1:**
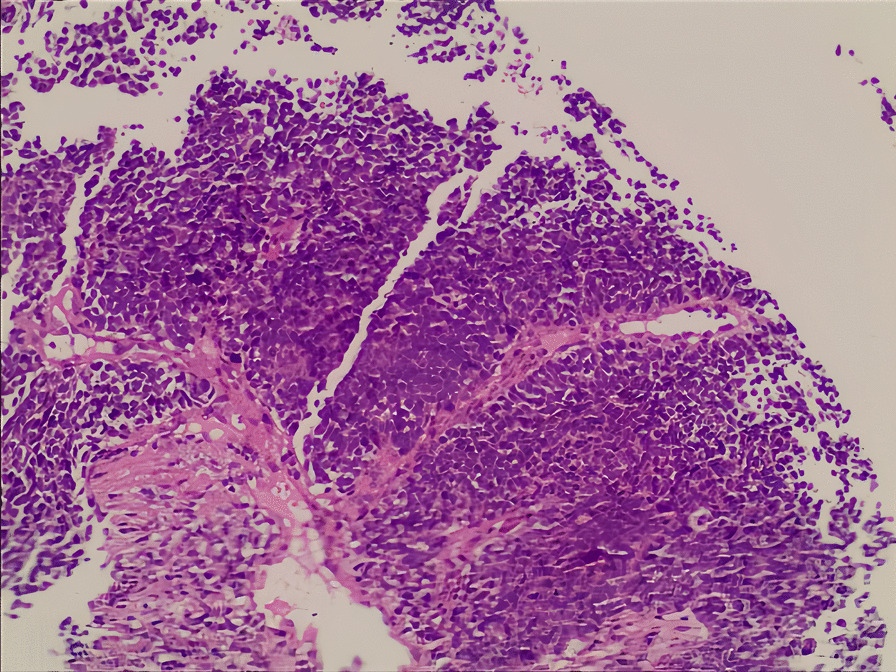
Lung lesion biopsy results of the patient. “Lung”: biopsy of small tissue, combined with histology and immunohistochemistry, to support the diagnosis of small cell carcinoma. Immunohistochemistry: P40* (−), P53* (Diffuse strong +), P16* (Diffuse strong +), CK* (−), K7* (−), CD56* (+), CgA* (+), SYN* (+), CD45* (−), Ki-67*(+ , about 80%)

**Fig. 2:**
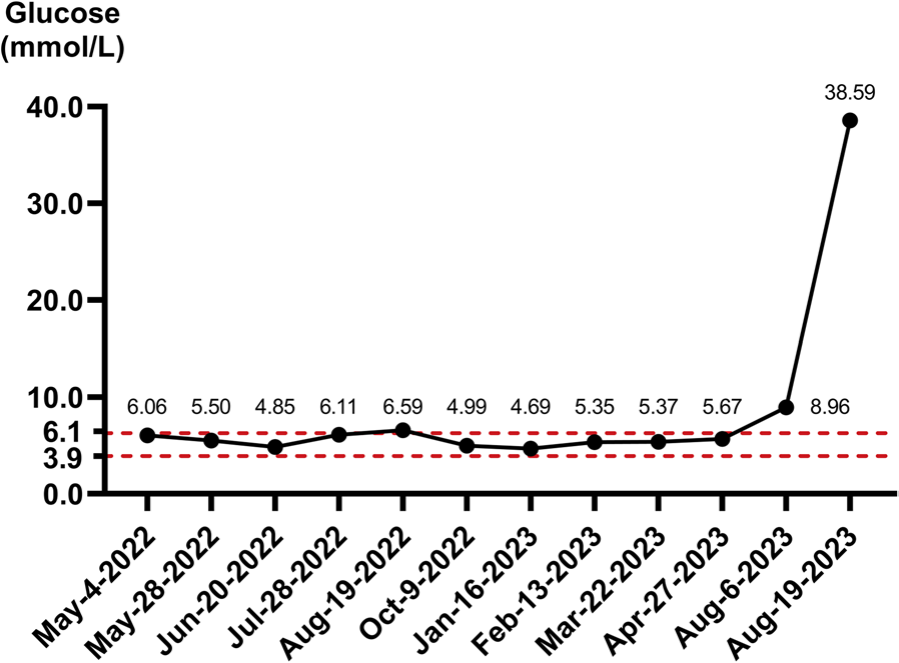
Blood glucose levels of the patient during the treatment process

**Fig. 3:**
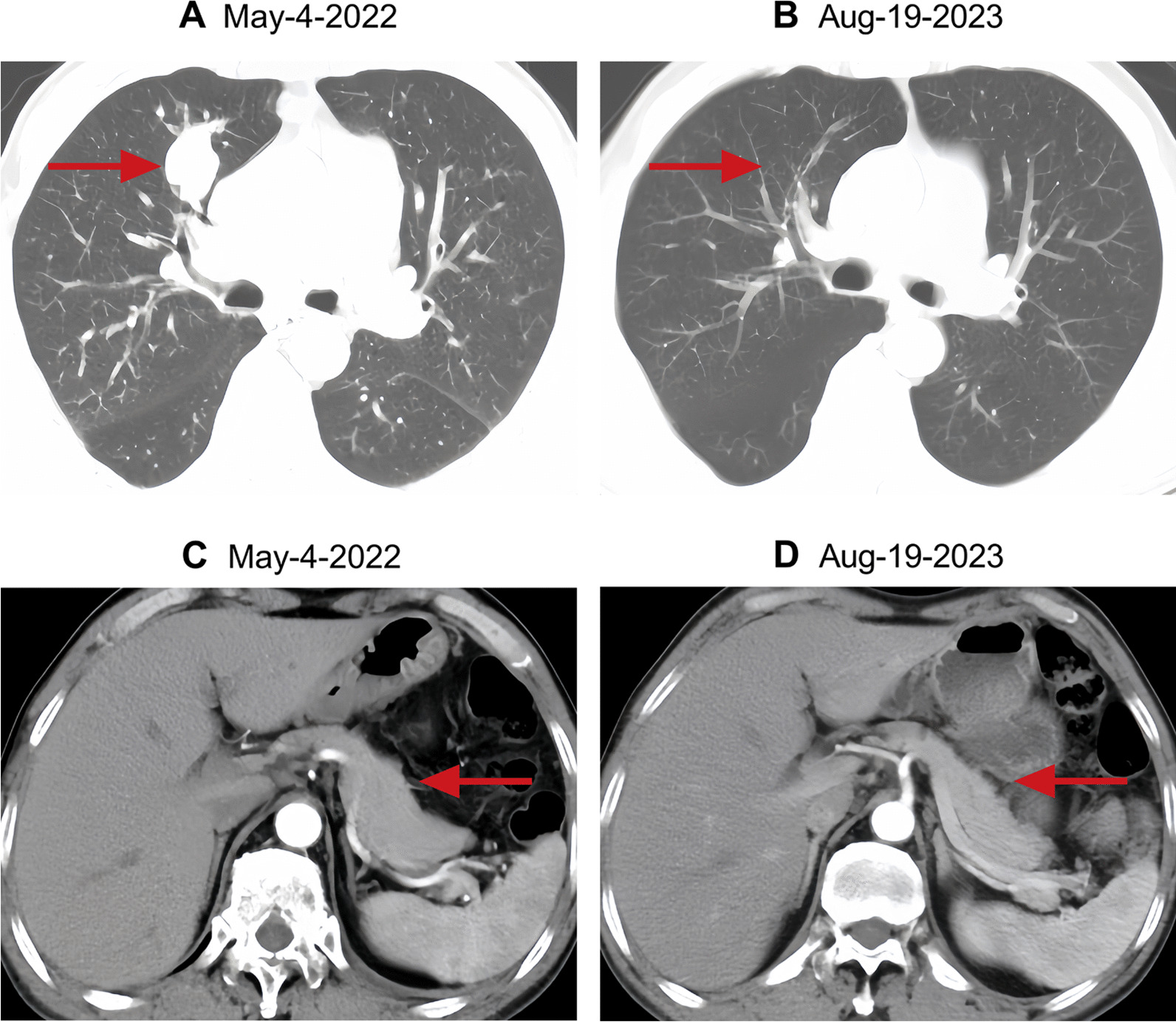
Computed tomography (CT) of the patient’s chest and abdomen during treatment. **A** Chest computed tomography imaging findings at the beginning of treatment. **B** Computed tomography of the chest of the patient with ICI-induced type 1 diabetes mellitus. Tumor lesions in **B** are significantly reduced versus those in **A**, as indicated by the arrows. **C** Abdomen computed tomography imaging findings at the beginning of treatment. **D** Computed tomography of the abdomen of the patient with ICI-induced type 1 diabetes mellitus. The pancreas in **D** does not show significant atrophy versus that in **C**, as indicated by the arrows

A 55-year-old male of Han nationality from China (height 1.70 m, weight 50 kg, body mass index 22.21 kg/m^2^) had no personal or family history of diabetes. He was initially admitted to the respiratory department of our hospital on 28 April 2022 due to respiratory symptoms of cough and chest pain. Relevant imaging studies were suggestive of lung cancer with multiple metastases to the cervical lymph nodes, brain, and bone. Further lung lesion biopsy (Fig. 1) confirmed the diagnosis of SCLC. On 04 May 2022, the patient received combination therapy of serplulimab, etoposide, and carboplatin for the first time for a total of six cycles. Tumor lesions were well controlled, and blood glucose monitoring was not indicative of an abnormal increase in blood glucose levels. After stopping therapy for more than 3 months, the patient continued to receive serplulimab alone on 16 January 2023, for a total of five cycles. The treatment process of the patient is presented in Table 1. On 19 August 2023, the patient presented to the emergency department of our hospital with a sudden onset of severe thirst, nausea, vomiting, deep respirations, and stupor. The test results were as follows: venous glucose was 38.59 mmol/L and glycosylated hemoglobin was 7.4%; pH was 6.95; base excess could not be calculated; bicarbonate was < 3.0 mmol/L; partial pressure of carbon dioxide was 12 mmHg; partial pressure of oxygen was 143 mmHg; lactic acid levels were 4.9 mmol/L; urine ketone was 2+ ; and urine sugar was 4+ . The patient was diagnosed with DKA and admitted to the endocrinology department of our hospital. The glucose levels of the patient during the treatment process are shown in Fig. 2, and computed tomography imaging findings of the chest and abdomen are shown in Fig. 3. After active blood sugar control, fluid supplementation, and correction of ketoacidosis, the patient’s condition began to stabilize. Further examination indicated that insulin and C-peptide release were extremely low (Table 2). Insulin autoantibodies and islet cell autoantibodies were not detected as diabetes-related autoantibodies, and the glutamic acid decarboxylase antibody (GADA) level was < 1.00 IU/mL. After excluding other drug factors, the diagnosis of ICI-T1DM was considered, and long-term insulin replacement therapy was required in the later stage.

**Table 1:** The patient’s treatment process

Treatment period	Period 1	Period 2	Period 3	Period 4	Period 5	Period 6	Period 7	Period 8	Period 9	Period 10	Period 11
Treatment date	04 May 2022	28 May 2022	20 Jun 2022	28 Jul 2022	19 Aug 2022	09 Oct 2022	16 Jan 2023	13 Feb 2023	22 Mar 2023	27 Apr 2023	06 Aug 2023
Treatment plan	HLX10 300 mg + CBP 400 mg + VP-16 360 mg	HLX10 300 mg + CBP 400 mg + VP-16 360 mg	HLX10 300 mg + CBP 400 mg + VP-16 360 mg	HLX10 300 mg + CBP 400 mg + VP-16 360 mg	HLX10 300 mg + CBP 400 mg + VP-16 360 mg	HLX10 300 mg + CBP 400 mg + VP-16 360 mg	HLX10 300 mg	HLX10 300 mg	HLX10 300 mg	HLX10 300 mg	HLX10 300 mg

*HLX10* serplulimab; *CBP* carboplatin, *VP-16* etoposide

**Table 2:** Oral 75 g glucose tolerance test results and C-peptide and insulin release results

Measurement time (min)	Glucose (mmol/L)	C-Peptide (ng/mL)	Insulin (mIU/L)
0	4.84	< 0.03	< 1.00
30	6.47	< 0.03	< 1.00
60	12.84	< 0.03	< 1.00
120	18.43	< 0.03	< 1.00

## Discussion

The most striking finding of this case report compared with previous reports is that ours is the first global report of an adverse event of ICI-T1DM with serplulimab. In addition, previous large-scale data studies found that ICI-T1DM usually occurred weeks to months after drug use, with a median of 10 weeks [5]. However, in our patient, T1DM did not occur until 68 weeks after receiving ICI treatment, which was a significantly longer time compared with that reported previously and a very rare occurrence per previous medical reports.

ICIs, as a milestone of antitumor therapy, have changed the management of cancer treatment. Although ICIs did not enter the market until 2011, they have been widely used in recent years, ranking second among the oncology products approved by the FDA [10]. ICIs do not directly kill tumor cells but rather counterbalance the tumor mechanism, reverse immune escape, activate immune response, and promote the killing of tumor cells by immune cells via remodeling T lymphocyte toxicity [11]. This makes the long-term survival of patients with advanced malignant tumors possible and provides new therapeutic indications in the early stage [12]. Two of the most prominent mechanisms currently used to block immune checkpoints are blocking the interaction between cytotoxic T lymphocyte-associated protein 4 and PD-1 and its ligand 1 (PD-L1) [13]. Although ICIs have greatly improved the prognosis of patients with tumors, their immune-related adverse reactions are attracting increasing attention.

Autoimmune endocrine gland adverse events caused by ICIs mainly include thyroid dysfunction and hypophysitis [14]; however, ICI-T1DM cannot be ignored. ICI-T1DM has some similarities with T1DM and fulminant type 1 diabetes mellitus [15]. Approximately 70% of patients suffer from DKA [6], which seriously threatens their lives if not detected and treated promptly. C-peptide level is generally low or even undetectable at disease onset. Fewer than half of the patients test positive for islet autoantibodies, among which GADA is the most common [6]. The patient in this report was not treated strictly according to the treatment cycle due to personal reasons such as treatment costs, family expenses, and work commitments. He received a combination of serplulimab, etoposide, and carboplatin for the first six cycles and only serplulimab monotherapy in the latter period for more than 6 months. The patient’s elevated blood glucose was time correlated with serplulimab use. No other drugs were used in the same period, suggesting serplulimab as the cause of type 1 diabetes; thus, the diagnosis of ICI-T1DM was considered. The patient was treated with ICIs for more than a year, and his blood glucose levels were always normal during the previous treatment period. Therefore, we did not pay enough attention to the slight increase in blood glucose levels (fasting venous blood glucose 8.96 mmol/L) in the last treatment before the occurrence of ICI-T1DM. Thus, less than 2 weeks after discharge, the patient was admitted to the emergency department due to sudden DKA. As the patient’s blood glucose levels were controlled after insulin treatment, subsequent treatment with serplulimab was not discontinued. The occurrence of ICI-T1DM in this patient was delayed. This is a condition often ignored by clinicians, resulting in severe adverse consequences to the patient. Accordingly, it warrants the close attention of clinicians. At the same time, we underscore the importance of interdisciplinary collaboration among oncologists, endocrinologists, and respiratory specialists when managing such complex cases.

The exact pathogenesis of ICI-T1DM is still unclear. The possible mechanisms are as follows: (1) Loss of immune regulation: ICIs enhance the antitumor immune response by inhibiting the PD-1/PD-L1 pathway, but this may lead to unintended adverse effects, including T1DM, as immune regulation may be lost [16]. (2) Islet β cell damage: inhibition of the PD-1/PD-L1 pathway leads to the activation of autoimmune T cells, increasing their infiltration and destruction of islet β cells [17], which may lead to insulin deficiency and ICI-T1DM. (3) Interferon (IFN) release: activated autoimmune T cells release IFNs in response to PD-1 inhibition, and these IFNs activate monocyte-derived macrophages [18], further damaging islet β cells. (4) Inflammation of the pancreas: patients with ICI-T1DM may show signs of pancreatic inflammation, including pancreatic atrophy, increased pancreatic enzyme levels, and peri-islet lymphocyte infiltration [17]. (5) Immune infiltration: immune cells may infiltrate the pancreas of patients with ICI-T1DM, leading to an increase in CD8^+^ T cells relative to CD4^+^ T cells and a lack of macrophages [19]. (6) Two-strike hypothesis: the development of ICI-T1DM may require two triggering events, that is, increased PD-L1 expression in stressed β cells followed by exposure to ICIs [20]. These factors may lead to the development of ICI-T1DM and not just the anticancer immune response.

It is worth noting that, to date, most cases of ICI-T1DM have occurred in high-risk patients with human leukocyte antigen DR4 (HLA-DR4) (76%), whereas other high-risk HLA alleles are not more common than those in the general population [21]. Until now, the risk could not be predicted based on family history or the presence of autoantibodies; thus, clinicians using these drugs should be aware of this side effect and appropriately reinforce patient health education. Moreover, some studies reported significant pancreatic atrophy after ICI-T1DM, but no pancreatic exocrine insufficiency was noted [22]. No pancreatic atrophy was found in our case most likely due to our short follow-up time. Long-term follow-up of this patient is therefore warranted.

## Conclusion

While using ICIs, it is important to be mindful of adverse events, mainly ICI-T1DM, in addition to monitoring its efficacy. The incidence of ICI-T1DM is low and not easy to identify; however, once it occurs, it endangers the life of patients from subsequent DKA. Moreover, even if hyperglycemia does not occur in the early and middle stages of ICI treatment, blood glucose levels and early signs and symptoms of diabetes must be monitored in patients in the later stages of treatment. Clinicians should remain vigilant throughout the treatment period to avoid irreversible consequences to patients.

## Data Availability

Data sharing is not applicable to this article as no datasets were generated or analyzed during the current study.

## Abbreviations

ICIs: Immune checkpoint inhibitors
T1DM: Type 1 diabetes mellitus
ICI-T1DM: Immune checkpoint inhibitor-induced type 1 diabetes mellitus
DKA: Diabetic ketoacidosis
CT: Computed tomography
GADA: Glutamic acid decarboxylase antibody
PD-1: Programmed cell death 1
PD-L1: Programmed cell death ligand 1
HLX10: Serplulimab
CBP: Carboplatin
VP-16: Etoposide
IFN: Interferon
HLA-DR4: Human leukocyte antigen DR4
